# Bringing the underground to the surface: Climate change stressors negatively affect plant growth, with contrasting above and belowground physiological responses

**DOI:** 10.1111/pce.14379

**Published:** 2022-06-21

**Authors:** Melissa A. Pastore

**Affiliations:** ^1^ Rubenstein School of Environment and Natural Resources University of Vermont Burlington Vermont USA; ^2^ Gund Institute for Environment University of Vermont Burlington Vermont USA

Terrestrial ecosystems sequester carbon from the atmosphere through a single biological process—photosynthesis—and thus considerable research has centered on how global change factors influence aboveground plant dynamics. For instance, it is well established that photosynthesis increases with temperature until reaching an optimum and then declines as temperature continues to rise (Berry & Bjorkman, 1980; Sage & Kubien, 2007), but that plants can also acclimate to maintain or increase photosynthesis in response to increased temperature (Way & Yamori, 2014). Low soil water availability, which can occur during drought or warming, is known to commonly reduce photosynthesis via stomatal closure, an adjustment that limits transpirational water loss (Chaves et al., 2003; Flexas et al., 2004). Our understanding of how global changes affect belowground plant carbon dynamics is much more limited, but studies of plant carbon allocation reveal the important role of belowground processes and signal the need for whole plant and system perspectives (e.g., Adair et al., 2009; Brunn et al., 2022; Chandregowda et al., 2022 in this issue; Hasibeder et al., 2015; Lyu et al., 2021; Pausch & Kuzyakov, 2018).

The carbon plants take in through photosynthesis has several potential fates, such as storage, growth, reproduction, the maintenance of existing tissues, plant defenses, root exudation and transfer to symbionts (Mooney, 1972). The partitioning of photosynthate among these fates, which is known as plant carbon allocation, impacts plant growth and function, plant carbon balance and soil organic carbon stocks, thereby feeding back to impact the global carbon cycle and climate change (Hartmann et al., 2020; Xia et al., 2017). Which carbon allocation pathways plants will prioritize under future environmental conditions and land‐use scenarios is not well understood, partly due to the difficulty in measuring carbon allocation. Studies often infer carbon allocation from biomass ratios (e.g., root:shoot ratios) and have revealed valuable insights (Poorter et al., 2012), but biomass ratios do not reliably relate to carbon fluxes like above and belowground respiratory losses or the partitioning of gross primary production (GPP) (Litton et al., 2007), dynamics that are needed for models but are harder to estimate (Ise et al., 2010).

In this issue of Plant, Cell & Environment, Chandregowda et al. report findings from a controlled environmental chamber experiment in which they determined how warming, drought and grazing affect plant carbon allocation via detailed measurements of leaf, root and whole‐plant carbon fluxes paired with measurements of above and belowground biomass. The authors focused on a globally occurring C_3_ perennial grass, *Festuca arundinacea* (tall fescue), which is widely used for pastures and forage. Importantly, they grew *F. arundinacea* plants in a carbon‐free potting mixture, which means that the carbon dioxide production they measured was derived from autotrophic respiration of recently assimilated carbon with minimal contribution from heterotrophic respiration. They exposed *F. arundinacea* to factorial temperature (26°C and 30°C) and water (well‐watered and −65%) treatments, and clipped plants halfway through the experiment to simulate grazing or harvesting.

Chandregowda et al. found that while both warming and drought strongly reduced plant growth and had negative effects on whole‐plant carbon fluxes, the mechanisms differed between climate stressors with important contrasting responses of above versus belowground plant physiology. Warming increased root respiration by 46%, potentially reducing carbon availability for growth, but did not affect leaf respiration or leaf net photosynthesis despite enhanced leaf nitrogen concentrations. Although leaf‐level physiology was insensitive to warming, there was no evidence that leaf photosynthesis or leaf respiration had acclimated to warming, but leaves may have been operating near the photosynthetic temperature optimum (Jacob et al., 2020). Differential temperature sensitivity between leaves and roots may help explain the contrasting responses of root and leaf respiration (Loveys et al., 2003). In addition to the important finding of distinct above versus belowground responses, the observation that warming‐induced reductions in biomass were not accompanied by declines in photosynthesis adds to a growing body of evidence that responses of plant carbon assimilation to global changes may not directly drive plant growth responses (Körner, 2015; Muller et al., 2011; Reich et al., 2018). Drought, in contrast to warming, reduced leaf net photosynthesis and increased the fraction of photosynthate allocated to leaf respiration. This drought‐induced reduction in leaf carbon balance was associated with decreased above and belowground plant biomass, but, unlike for warming, drought increased the fraction of total plant biomass allocated belowground, an adjustment to optimize soil resource uptake during drought (Poorter et al., 2012).

Chandregowda et al. additionally addressed the question of how grazing or harvesting of aboveground biomass, which is common in pastures and rangelands, can alter plant carbon fluxes and allocation. Very few studies have investigated whether grazing affects carbon allocation to root respiration, with some finding positive effects and others finding no effect (Bahn et al., 2006; Holland et al., 1996; Thorne & Frank, 2009). Chandregowda et al. found that harvesting more than doubled belowground respiratory allocation, altering the functional balance between above and belowground plant tissues. Additionally, harvesting during warming or drought reduced plant regrowth by 35% and 77%, respectively. These results suggest that some pasture management practices may exacerbate the negative effects of climate change, particularly reduced rainfall, on pasture production and grazing resources.

**Figure 1:**
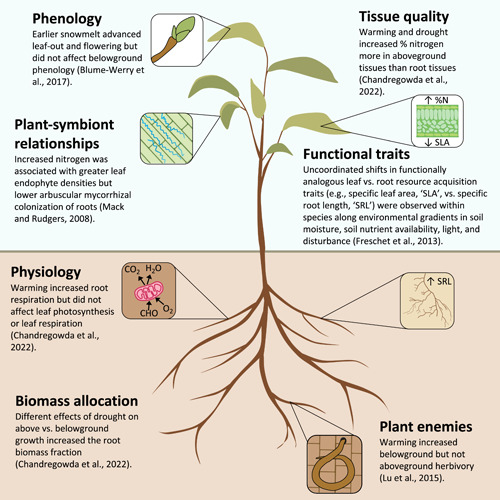
Examples of differing above versus belowground plant responses to various global changes. These examples highlight responses of phenology, plant‐symbiont relationships, physiology, growth rates, tissue quality, functional traits and interactions with plant enemies, and there are likely additional plant phenomena that respond in contrasting ways above versus belowground. Studies exclusively assessing aboveground responses may miss important belowground responses and vice versa. [Color figure can be viewed at wileyonlinelibrary.com]

These distinct responses of different plant components to warming and drought highlight the importance of studies that jointly consider above and belowground responses to global changes. While research in this area is limited, studies are finding differences in responses of diverse above versus belowground phenomena to a variety of global changes (Figure 1). Chandregowda et al. highlighted different above versus belowground responses of plant physiology, growth rates and tissue quality in response to climate stressors (Figure 1). In accordance with those results, drought has been shown to reduce the growth metabolism of shoots while increasing that of roots (Gargallo‐Garriga et al., 2014). Beyond responses of plant physiology and metabolism, experiments demonstrate that above versus belowground phenology can respond asynchronously to climate change. For instance, snow manipulations in heath and meadow communities showed that earlier snowmelt advanced leaf emergence and flowering but did not affect the timing or amount of root production despite warmer soil temperatures (Blume‐Werry et al., 2017) (Figure 1). Studies have also revealed that above versus belowground plant‐symbiont and plant‐enemy relationships may have different sensitivities to climate change (Lu et al., 2015; Mack & Rudgers, 2008) and that responses of some intraspecific above and belowground functional traits may be uncoordinated (Freschet et al., 2013) (Figure 1). Thus, global changes can asynchronously affect the above and belowground plant dynamics that jointly influence whole ecosystem responses.

Together, these studies indicate that both above and belowground processes should be represented in models that predict plant responses to global change, but additional studies are needed to provide process‐level knowledge. How differing above versus belowground physiological and carbon allocation effects scale to whole canopies or ecosystems and over time is unclear. Additionally, further studies are needed to determine how climate change may differentially affect above and belowground plant responses across species, plant functional types, and developmental stages, and how shifts in carbon allocation influence long‐term soil carbon storage. How climate change impacts root carbon export, which affects soil organic matter, is particularly poorly understood, but advances in isotopic methods and in‐situ root exudation measurements make it possible to quantify and upscale rhizodeposition (Brunn et al., 2022; Pausch & Kuzyakov, 2018; Phillips et al., 2008). Root carbon dynamics could be especially important to investigate in systems like grasslands and arctic tundra where a majority of plant biomass occurs belowground (Iversen et al., 2015; Pastore et al., 2021). Approaches like that used by Chandregowda et al. pairing temporal biomass and carbon flux measurements in a whole‐plant context will advance process‐level understanding of plant carbon allocation in response to global change. In addition to warming and drought, we need a greater understanding of the sensitivity of carbon allocation to other global change pressures and interactions among those pressures, such as elevated carbon dioxide, pulse heat events, fire disturbance, insect outbreaks, nitrogen deposition, freeze‐thaw cycles, ice storms and rain‐on‐snow events. Considering how multiple environmental changes influence both above and belowground plant dynamics will allow for improved predictions of carbon feedbacks to the atmosphere and better preparation for Earth's future.
